# Disease Prevention: An Opportunity to Expand Edible Plant-Based Vaccines?

**DOI:** 10.3390/vaccines5020014

**Published:** 2017-05-30

**Authors:** Christopher Concha, Raúl Cañas, Johan Macuer, María José Torres, Andrés A. Herrada, Fabiola Jamett, Cristian Ibáñez

**Affiliations:** 1 Departamento de Biología Marina, Universidad Católica del Norte, Programa de Doctorado en Biología y Ecología Aplicada, Coquimbo 1780000, Chile; christopher.concha@ucn.cl; 2 Departamento de Ingeniería en Alimentos, Universidad de La Serena, Programa de Doctorado en Ingeniería en Alimentos y Bioprocesos, La Serena 1700000, Chile; raulhcs@gmail.com (R.C.); mtorresossandon@gmail.com (M.J.T.); 3 Departamento de Biología, Universidad de La Serena, Programa de Doctorado en Ingeniería en Alimentos y Bioprocesos, La Serena 1700000, Chile; johanmacuer@gmail.com; 4 Facultad de Salud, Universidad Autónoma de Chile, Talca 3460000, Chile; andresherradah@gmail.com; 5 Departamento de Química, Universidad de La Serena, La Serena 1700000, Chile; fjamett@userena.cl; 6 Departamento de Biología, Universidad de La Serena, La Serena 1700000, Chile

**Keywords:** edible vaccine, medicinal food, genetic modification, immunogenicity, disease outbreaks, food biotechnology

## Abstract

The lethality of infectious diseases has decreased due to the implementation of crucial sanitary procedures such as vaccination. However, the resurgence of pathogenic diseases in different parts of the world has revealed the importance of identifying novel, rapid, and concrete solutions for control and prevention. Edible vaccines pose an interesting alternative that could overcome some of the constraints of traditional vaccines. The term “edible vaccine” refers to the use of edible parts of a plant that has been genetically modified to produce specific components of a particular pathogen to generate protection against a disease. The aim of this review is to present and critically examine “edible vaccines” as an option for global immunization against pathogenic diseases and their outbreaks and to discuss the necessary steps for their production and control and the list of plants that may already be used as edible vaccines. Additionally, this review discusses the required standards and ethical regulations as well as the advantages and disadvantages associated with this powerful biotechnology tool.

## 1. Introduction: The Current State of Vaccination

Infectious diseases account for more than 54% of total mortality in developing countries, where vaccines are the most effective means of prevention [1,2]. Vaccination is a relatively new process that was introduced approximately 200 years ago with the purpose of delivering a minimum dose of a disease-causing pathogen to induce a humoral or cellular immune reaction without subjecting the individual to the risk of true infection [3,4,5,6]. Diseases such as cholera, typhoid fever, tuberculosis, and poliomyelitis have been controlled by massive vaccination campaigns [7].

Vaccine development is currently in an extremely dynamic phase, and vaccines are safer and more effective than ever. However, to achieve the public health goals set by the World Health Organization (WHO) for 2011–2020 [8], more people must benefit from vaccines that save lives and prevent old and new diseases. In most countries, routine immunization programs now go beyond the traditional six childhood vaccines against diphtheria, tetanus, whooping cough, measles, polio, and tuberculosis [8]; vaccines to prevent hepatitis B, rubella, pneumococcal disease, and rotavirus have been included in several traditional vaccination programs worldwide [8,9]. Over the next decade, it is expected that a growing number of countries will use new vaccines, such as the human papillomavirus (HPV) vaccine [9]. 

Despite the advantages of vaccination, limitations restricting the use of vaccines remain. Not all pathogenic agents can be cultivated in an exogenous medium and, due to their highly pathogenic features, the cultivation of some agents demands biosecurity and biosafety infrastructures that are difficult for some countries to afford. Consequently, the production of certain vaccines remains costly and restricted in numerous countries, thus generating an undesirable dependence on hygiene [9]. Another restrictive factor is that, although the attenuation of bacteria or viruses involves very controlled processes, the possibility that these pathogens could revert to their original pathogenic form must be considered [10]. Additionally, the highly specified expiration time and refrigeration requirements inherent to nearly all commercial vaccines demand constant attention to the pathogen contained in such vaccines, thus increasing control, storage, and distribution costs [7]. Vaccine degradation after acid digestion in the stomach is another concern [11].

Recognizing these limitations and exploiting advances in recombinant DNA technology, Mason et al. succeeded in expressing a surface antigen from hepatitis B in tobacco plants [12]. This finding immediately suggested that plants were potentially effective vectors for the production of vaccines to prevent diseases, giving rise to the concept of “edible vaccines,” a term coined in 1990 by Charles Arntzen [13]. However, the development of edible vaccines remains in its infancy, and various medical, legal, ethical, and environmental uncertainties have emerged [7]. The aim of this review is to provide an overview of edible vaccines to assess their potential as real functional foods for helping to control and prevent pathogenic diseases and outbreaks in remote areas and on longer time scales. Moreover, this overview discusses the types of edible vaccines, how they are developed and evaluated, their advantages, disadvantages, challenges for their producers, consumer populations, and vaccine distributors. Finally, because most edible vaccines currently being developed and tested in animals are not for diseases associated with recent outbreaks, the feasibility of expanding edible vaccines studies to those diseases with more recent outbreaks is discussed.

## 2. The Problem of Infectious Diseases and their Outbreaks

In the early twentieth century, infectious diseases caused by pathogenic microorganisms were the main source of mortality worldwide [6,14]. Although the lethality of infectious diseases has decreased due to the use of different control agents and the application of sanitary measures, such as vaccination [6,7], recent outbreaks of pathogens have occurred in different parts of the world [15,16,17]. These outbreaks are associated with the relaxation of certain levels of hygiene control; overcrowding of cities, which tends to perpetuate certain diseases; the presence of factors that decrease the ability of each individual to confront pathogens; and the growing mobility of the world’s population to locations where infectious diseases have not existed previously [18], causing unexpected consequences for the entire global health system. For example, after 25 years without any positive case, an outbreak of measles recently occurred in Chile [19]. Another measles outbreak occurred in the United States, with 700 cases in 2014 and 171 cases in 2015 [17]. In both of these outbreaks, a person who returned from a country where the disease is active was the triggering factor, revealing the new threat associated with aerial mobility. Moreover, in recent years, increasing vaccine-hesitant parents has risen around the world for diverse reasons, ranging from objections to buying pharmaceutical products to religious ideologies or simply as a fashionable practice [20]. It is particularly instructive to observe a recent case of a Spanish child who was infected with diphtheria (*Corynebacterium diphtheriae*), a disease considered “extremely rare” in Spain that is still circulating in Russia and other former Soviet republics [16,21]. In this case, the parents had voluntarily decided not to vaccinate their two children against this bacterium, citing misinformation about the harms of vaccination in children [22]. Outbreaks of infectious diseases observed in the last decade have not only occurred in countries with food and health requirements that historically have been affected by this phenomenon but are also increasingly affecting countries with consolidated health systems [8], demonstrating the need for effective prevention strategies to stop the worldwide emergence of new pandemics and serving as a reminder that only one disease has been eradicated so far: smallpox.

Table 1 shows the most recent outbreaks of diseases occurring around the world since 2010, highlighting the number of countries affected by these outbreaks, years of outbreaks, and edible vaccines already tested in animals. Zika virus was the most mobile disease in terms of countries, affecting 29 nations in the last six years (Table 1). Only three diseases with recent outbreaks have an edible vaccine that has been tested in animals but not yet in humans. A more detailed description of disease outbreaks that have occurred since 2010, divided by continents, epidemiological agents, and time of year of the outbreak, is presented in Supplementary Materials Table S1.

The limited number of edible vaccines developed for recent outbreaks raises questions about whether it is time to expand edible vaccine studies to those diseases with more recent outbreaks, which warrants a deeper investigation of the current state of edible vaccines.

## 3. Edible Vaccines: What Are They and How Do They Work?

The information outlined above highlights the importance of identifying novel, rapid, and concrete solutions for control and prevention. Edible vaccines are of interest as alternative methods of vaccination; as the name suggests, these are foods that provide nourishment in terms of vitamins, proteins, and other nutritional qualities that also act as vaccines to immunize the consumer against a certain disease.

Edible vaccines include all vaccines that are produced in a type of edible format (i.e., part of a plant, its fruit, or subproducts derived from that plant) that, upon oral ingestion, stimulate the immune system [5,7,30]. It is worth mentioning that edible does not necessarily mean nutritious, tasty, or organoleptically pleasing, since edible vaccines need only be safe (non-toxic) for human consumption.

**Figure 1:**
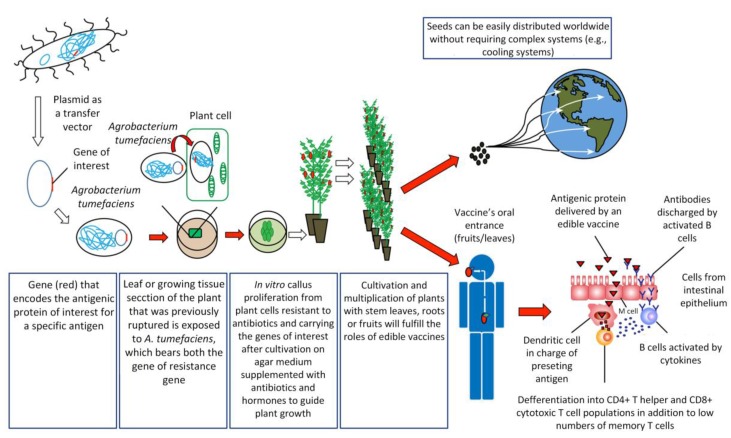
Procedures involved in obtaining an edible vaccine and an immune response. Edible vaccine development begins with the identification of the gene encoding the antigenic protein and its introduction into the plant that will process the food (edible vaccines), which can then potentially be distributed globally. After an edible vaccine has been consumed, and the subsequent passage of the antigenic protein through the M cells specialized in the delivery of antigens to dendritic cells, the individual’s immune system triggers a response involving B cells and T helper cells as the main factors. For simplicity, other routes of antigen delivery have been omitted. This figure was adapted from the work of Langridge [4].

To create an edible vaccine, the information necessary to produce an antigenic protein must be introduced into the plant of interest by genetic engineering techniques (Figure 1). Once an individual consumes an edible vaccine, the outer wall of plant cells protects the antigens from degradation by gastric secretion, allowing the antigens to be delivered to the intestinal mucosal surfaces, where they are absorbed by different mechanisms in order to stimulate a strong and specific immune response [31].

One of the main routes of antigen capture at the intestinal level is through Microfold (M) cells. M cells represent a small number of specialized follicular-associated epithelium (FAE) enterocytes found primarily in the gastrointestinal tract. These cells efficiently capture a wide variety of macromolecules and microorganisms from the lumen of the small intestine to submucosal antigen-presenting cells (APCs) on Peyer’s patches [32]. Among APCs, dendritic cells (DCs) are the most potent antigen-presenting cells in priming naïve T cells to initiate an adaptive immune response [33]. DCs in steady state are found in an immature stage, characterized by high endocytic activity and a low capability to prime naïve T cells. However, under inflammatory conditions, DCs mature, increasing the expression of co-stimulatory molecules and migrating to T-cell-rich zones in lymph nodes. There, they present antigens together with the release of cytokines facilitating the differentiation of naïve antigen-specific T cells into effector cells and their migration to the specific site of inflammation [34]. Interestingly, intestinal DCs can promote the activation of naïve T cells and the differentiation to follicular T helper cells (Tfh) either by directly promoting Tfh differentiation or indirect by promoting Th17 cells that later will become Tfh [35,36]. Tfh cells specifically promote the activation of follicular B cells and the generation of IgG and IgA-secreting plasma cells [37]. Then, these activated B cells leave the lymphoid follicles and migrate to the mucosa associated lymphoid tissue (MALT), where plasma cells secreting immunoglobulin A (IgA) antibodies are found [4,38]. These IgA antibodies are transported across epithelial cells in secretions to the lumen, where they can interact with antigens [38]. It has been recently shown that DCs are critically important in IgA class switching and secretion in B cells [39]. Moreover, DCs can directly capture luminal antigens by projecting dendrites through the epithelial cell layer and into the lumen [40]. Another recent mechanism of antigen capture in the small intestine involved goblet cells, a cell type involved in the production of mucins. By intravital microscopy it was shown that goblet cells can directly capture and deliver antigens to intestinal DCs [41]. An efficient, edible vaccine will stimulate specific T and B cell responses, which will promote long-lasting memory cells for subsequent encounters in which the antigen is presented in the course of an actual infection [4,38]. However, one of the debates about the oral administration of vaccines has been the development of “oral tolerance”, referring to the phenomenon mediated by T cells that involves a decrease in the specific immune response to antigens previously encountered through the oral route [42,43]. In the intestinal immune system, the release of antigens occurs in the absence of inflammation (because antigen presentation is not mediated by adjuvants that induce this inflammation), where the antigens are presented to T cells by immature dendritic cells, inducing tolerance [44]. This occurs by the secretion of cytokines, such as IL-10, or by direct cell-to-cell contact, where regulatory T cells interfere with the maturation of dendritic cells, altering their tolerogenic function [44]. Repeated administration of antigens in the mucosa may even result in the suppression of the humoral immune response [45], and it remains difficult to generate vaccines with stable concentrations of antigen in transgenic plants. Recent studies have applied different strategies to overcome this problem. For example, Kesik-Brodacka et al. use hepatitis B virus (HBV) core protein (HBcAg) as a carrier of the antigen to induce immunogenicity, with promising results [46]. Other strategies involve intramuscular priming before the delivery of the edible vaccine [47]. However, more studies are necessary to efficiently overcome this problem. Alternatively, these particular issues provide the basis for the introduction of edible vaccines in solving problems of autoimmune diseases, based on the selective activation of the autoimmune system to teach the body to tolerate antigenic proteins [38]. Therefore, oral administration of autoantigens could induce tolerance [44].

### How Are Edible Vaccines Developed?

The mechanisms of edible vaccines involve a series of general principles. The first step consists of the identification, isolation, and characterization of the antigen of interest [48,49]. This antigen must elicit a strong specific immune response [7,30,50]. If the latter criterion is met, the gene encoding for this antigen must be cloned into a transfer vector carrying an antibiotic-resistance gene, followed by transformation of the plant of interest. Plant viral vectors appear to be the most promising for expressing foreign proteins in plants [51]. Plant transformation is attained by different methodologies [13,52]. One of the most commonly used methods for efficiently transferring recombinant DNA into plant cells involves the bacterium *Agrobacterium tumefaciens* [4,52,53]. An *Agrobacterium* strain has been designed to eliminate virulent genes that produce a tumor growing at the base of plants while retaining the genes involved in efficient DNA transfer. The tumor DNA (T-DNA) is contained in a plasmid called the Ti plasmid [53]. The sequence of interest (pathogen) is then inserted into T-DNA to produce the antigenic protein [7,53]. Once the transgene (T-DNA + antigen DNA) is integrated into the plant genome, the sequence should be expressed and inherited in a typical Mendelian fashion [54,55], following permanent or temporary (transient) expression of the antigen of interest in the plant or fruit [50]. Later, this genetic line may be propagated by vegetative methods (cutting) or seeds arising from asexual reproduction [56]. This technology is time-consuming, and the scientific infrastructure costs can be a barrier for massive production, especially in low-income countries [57,58,59]. However, transient transformation using either *Agrobacterium* or viral vectors is robust, less time-consuming, easier to manipulate, and offers better opportunities for the industrial production of vaccines or vaccine-related products in a short time [57]. A limitation of transient transformation is that transformation must be repeated if new plant products are required [57,58]. Ultimately, both transformation systems have their advantages and disadvantages, and the selection of one of these systems depends on the long-term aims and/or urgency of implementing vaccination.

However, the genetic transformation process is not a trivial event. Some agronomically important species (for example, soybeans and most cereal grains) strongly resist *Agrobacterium* transformation. For such plants, a bioballistic method (micromissile bombing) is commonly used, in which gold microparticles are coated with DNA and then blasted into the vegetables using compressed helium gas to attain random transgenic incorporation into the target plant’s chromosomal DNA [54]. Due to the random nature of the insertion, there is variability in the percentage of the genetic transformation achieved, and post-transformation diligence is required to select the most vigorous and stable transgenic lines.

Bioballistic methods are also a very efficient alternative when the objective is the plant chloroplast, since more than one copy of the gene of interest can be integrated, thus improving the efficiency of protein expression [60,61]. In addition, because plastids are not contained in the pollen of most plant species, public acceptance of chloroplast-based transformation seems promising [62]. As mentioned above, edible vaccines can also be generated using viral vectors for expression, by infecting a plant with a virus that is able to replicate independently and transcribe and translate a recombinant protein inserted into the virus genome that corresponds to a characteristic epitope of another pathogenic agent, whether it be from animals or humans [61,63,64]. The system is very efficient [53,61,63] since the soluble protein is not only expressed in the host plant cells but may also be fused to the capsid of the virus and multiply each time the virus replicates [61]. One of the first edible vaccines developed using the viral vector methodology was a virion that expressed malarial epitopes on its surface [63,65]; other viruses that have been used include the potato virus, the bamboo mosaic virus, the papaya mosaic virus, and the cowpea mosaic virus [51,63,66]. The final step is the oral administration of the vaccine, whether through direct consumption of the part of the plant that contains the vaccine or by ingesting the part of the plant that carries the vaccine in concentrated pill form. However, as we discussed in the previous section, immune tolerance is a potential problem for edible vaccines, and thus, in order to overcome this immune tolerance, increased concentrations of antigen are needed in the vaccine to stimulate a strong immune response [3,67]. In fact, studies in the potato in 2005 showed that, although vaccine parenteral administration requires a dose of 40 µg of HBsAg (surface antigen of hepatitis B), oral vaccines require at least three doses of 100 g of potatoes containing a dose of 1 mg of HBsAg to be partially effective [63]. Better results have been obtained through production by viral vectors of up to 295 µg of protein in 1 g of fresh weight of plant tissue [68]. 

Due to the difficulty of achieving stable vaccine production, compound systems have been developed to generate more stable protein concentration yields in plant tissue systems. These systems combine the integration of *Agrobacterium* DNA with high protein expression of the plant RNA virus and posttranslational capabilities of a plant; this system is called a “launch vector” [69]. In this system, the β-1-3 1-4 glucanase (lichenase) thermostable enzyme, which is stable up to 65 °C, is used as a carrier to enhance stability and protein expression [69,70]. Transient expression using vector methodologies based on viruses or agroinfection in specific parts of a plant can facilitate stabilization via convenience and speed; both viral vectors and systems based on *Agrobacterium* infiltration can produce large amounts of protein in the days after the initial molecular cloning event, in contrast to months for the development of plant and transgene expression [63,71]. The system based on viral vectors not only enables the expression of the antigenic protein in the particular plant tissue more quickly and efficiently but also results in higher protein concentrations due to expression of this protein in the virus structure that is replicated [72]. First-generation viral vectors retain infectivity in the plant but have raised safety concerns. Second-generation viral vectors maintain a minimum of viral elements required for replication of the vector, and most DNA delivery to the target plant is via non-viral elements [73]. The latter are called viral “deconstructed” vectors and deliver higher performance than the full virus [73,74,75]. These types of vectors have been used as an expression system for monoclonal antibodies due to their high and stable levels of protein expression in plant tissue [76,77,78]. An example of this is the production of antibodies for West Nile virus in *Nicotiana benthamiana* developed by agroinfiltration [77].

## 4. Edible Vaccine Advantages and Disadvantages

During the past 10 years, many studies have been conducted regarding the potential to express antigens in the edible parts of plants, with very promising results [62,79,80,81,82,83,84,85,86,87]. It appears possible that this type of oral immunization may become a realistic main strategy in significantly preventing devastating diseases, particularly in low-income countries [13]. Moreover, edible vaccines do not require an extensive framework for their production, purification, sterilization, packaging, or distribution, reducing costs in the long term compared to traditional vaccines [7,50,88,89]. Furthermore, the distribution and maintenance of the vaccine are easier than for conventional vaccines, enabling application of a form of immunization worldwide without the constant cold chains used to preserve conventional vaccines [10,38]. Consumption of a raw material is another advantage of plant-based vaccines that reduces the cost of processing and purification of antigens [90] as well as the potential degradation of antigens by the gastrointestinal tract due to the protective role of plant cells inside the stomach [91]. Antigen expression in seeds allows maintenance and stability for longer periods, another advantage of edible vaccines [91].

Although edible vaccines are presented as a lower-cost option from a strategic point of view after production of the transgenic plant, this statement is not strictly true. While the administration of an edible vaccine is less complex than conventional vaccine administration because of the use of the oral route, the costs associated with the development and distribution of edible vaccines is a complex issue, particularly for the storage and maintenance of transgenic plants [92,93,94]. Additionally, control, purification, and biosafety are the responsibility of pharmaceutical companies, which involves additional costs and presents a barrier to the development of vaccines by small- and medium-size pharmaceutical companies [95]. In that sense, edible vaccines appear to be more promising in terms of animal vaccination [4,5,96], although the quality and safety of raw plant materials need to be assured. Another limitation of edible vaccines is the uncertainty related to the calculation of adequate oral administration dosage, which may require several rounds of administration, increasing the final cost of its application [97,98]. As long as the production costs remain high and a proper estimation of necessary antigen concentration remains unresolved, the future of edible vaccines will be as uncertain as that of traditional oral vaccines. 

Despite these issues, the potential of edible vaccines for immunization is undisputed. A notable example is the outbreak of Ebola virus in Africa in mid-2014, which caused a great number of casualties. No vaccine or globally tested treatment against Ebola virus is available [15]. *Nicotiana benthamiana* plants were used to transiently express three monoclonal antibodies that recognize Ebola virus surface glycoproteins isolated from individuals who survived Ebola infections [99], demonstrating that plants can be effectively used as biopharmacies. The development of an edible vaccine against this lethal disease would be extremely helpful (once the viral antigen that triggers an effective immune response has been identified) in regions where the transportation and delivery of conventional vaccines are difficult. The goal would be to deliver not only vaccines but also “pharmafood”. The objective in creating a vaccine as a food is to create a food source to reinforce health, particularly in underdeveloped countries, where it is difficult to obtain treatments that require complex equipment for their development or conventional vaccines that are difficult to store and transport. However, increased developmental research is essential, as is the need to develop essential legislation as soon as possible, before mass production occurs. Among other concerns, overconsumption of these plants bearing antigens that stimulate the immune system might produce overstimulation of the immune system itself. Moreover, the secondary effects of antigen ingestion should be more thoroughly investigated over the long term, similar to the production of traditional vaccines [100]. Another important factor to be considered is the site where edible vaccine-producing plants are grown. Absolute control should be exercised to protect the environment where such plants are grown to avoid the loss of seeds or pollen during plant removal. The presence of pesticide residues and secondary or toxic metabolites in the plants may pose a major problem [13]. Post-production of the transgenic plant, the risks associated with the use of this plant and its crop are directed to the spread of pollen, seed dispersal, possible horizontal gene transfer, and protein toxicity in herbivores [53]. Contact with insects and release of contaminated water into the environment are also possible mechanisms of transgene escape, though the escape of genes into a food chain is a more serious concern that cannot be underestimated. However, the likelihood and severity of each risk depends on the plant species and the antigen for each vaccine in transgenic plants [73]. Another important point is that, although, in principle, the development of an edible vaccine has been presented as a solution for the stimulation of the immune response based on the ingestion of a portion of a plant, the process presents difficulties in standardizing antigen concentrations in different plant tissues [48]. The prime difficulty lies with the plants’ inherent genetic variability, even in plants propagated by in vitro asexual conditions (e.g., somaclonal variation). Here, factors such as growth and fruit development, type ,and texture of eatable leaves or roots might influence the availability of antigens [101]. Nonetheless, future prospects also include the possibility of generating vaccines in unicellular green algae, which have many of the same advantages as land plants but much simpler handling and faster mass production [102]. Commercialization of edible vaccine-producing plants might face problems in countries that do not allow transgenic food sales or are not willing to allow the entry or consumption of plants (or parts of plants) that produce edible vaccines. However, the pros and cons of edible vaccines are not restricted to legislation and distribution, as shown by Jacob et al. [96] and Waheed et al. [62], who have presented general summaries of the advantages and disadvantages of edible vaccines. 

## 5. Plants Already Transformed for Use as Edible Vaccines

Most plants studied as edible vaccines have been transformed to express antigens for rotavirus, cholera, gastroenteritis, autoimmune diseases, or rabies [53]. Additionally, most studies have used potatoes for cultivation, but potatoes may not be the best choice for edible vaccines because cooking or boiling may destroy most of the antigenic proteins. Other plants, such as tomatoes, corn, tobacco, bananas, carrots, and peanuts, have a more promising future as edible vaccines, not due to their widespread use but due to the successful development and testing of genetic transformation methods [7,53].

The plant checklist that follows presents developed edible vaccines that have already been tested in animals and whose use is expected to be authorized in both human and animal medicine. A summary of this checklist is presented in Table 2.

### 5.1. Potatoes

Mason et al. conducted the first assay based on a vaccine produced in potatoes (*Solanum tuberosum*) to combat enteritis produced by *Escherichia coli* strain LT-B in mice [103]. That same year, the effectiveness of antigens produced by potatoes against the pathogen from Norwalk virus capsid and the non-toxic subunit (CT-B) of *Vibrio cholerae* enterotoxin was demonstrated in rats and human volunteers [27,104]. Tacket’s second-phase clinical assay (Phase I considered patients previously vaccinated) involved the study of human immune responses to Norwalk virus capsid expressed in potatoes, with 95% (19 out of 20 volunteers) developing a type of immune response. However, a significant increment was not always obtained [56]. In 2005, Thanavalas’s group proposed that the potato might have a role as an oral reinforcement to the hepatitis B injectable vaccine in humans [85]. Moreover, edible vaccines have also been developed as an oral reinforcement to injectable vaccines for animal protection. For example, an edible vaccine was developed in potatoes to protect minks from diseases caused by mink enteritis virus (MEV) [105]. In wild rabbits (*Oryctolagus cuniculus*), immunization via potatoes producing the protein VP60 provided protection against infection produced by rabbit hemorrhagic virus (RHDV) [106].

### 5.2. Tobacco

First, we want to highlight that tobacco per se is not an edible plant; rather, it is used as a proof-of-concept model species for edible vaccine development. Thus, in 1996, in parallel with the potato studies, transgenic tobacco (*Nicotiana benthamiana)* plants expressing a protein from Norwalk virus capsid that produces gastroenteritis were developed [50,53,107] and resulted in antibody, specifically IgA and IgG, development in rats [50,108]. In 2007, transgenic tobacco expressing the virus VP1 protein from chicken infectious anemia was reported [109]. Other studies in tobacco have demonstrated the ability to express a polypeptide associated with hepatitis B [50]. In this study, it was feasible to stimulate a humoral immune response that produced the HBsAg; such stimulation evoked higher blood T-cell counts, and these results were used to calculate correlations of the immunoglobulin A and G humoral responses with the corresponding vaccine dose [50,110]. Gómez et al. [111] endeavored to more effectively express the virus antigen in transgenic tobacco. In 2012, transgenic tobacco plants expressing HPAIV H5N1 from avian flu virus gave rise to IgG stimulation when tested in rats [112,113]. Recently, transgenic tobacco plants expressing a protein from *Eimeria tenella*, the agent that causes coccidiosis [84], and transgenic tobacco plants to combat anthrax [114] were reported. In the latter, the tobacco expressed a protective antigen (PA) that resulted in elevated serum IgA and IgG in murine models.

### 5.3. Tomatoes

An effective vaccine candidate against the coronavirus that causes a highly acute respiratory syndrome (SARS) was developed in the tomato (*Solanum lycopersicum*), [23]. A study in 2006 showed that tomatoes expressing the Norwalk surface virus protein that were dried outdoors instead of lyophilized before consumption by rats provided immune protection superior to that of potatoes [50,115]. Tomatoes have also been used to express CT-B protein from *Vibrio cholerae* B toxin, as supported by ELISA and Western blot analysis in leaves, stems, fruits, and other tissues [28]. HBsAg has recently been produced in tomatoes of the Megha variety, as confirmed by qRT-PCR and ELISA, the first report of stable expression of an antigen in tomatoes [50,116,117]. In 2008, human beta-amyloid was expressed in the tomato as a potential vaccine against Alzheimer’s disease [118]. Another study of transgenic tomatoes included the fusion antigen F1-V from *Yersinia pestis*, a bacterium that is highly pathogenic and causes pneumonic, septicemic, and bubonic plagues [79]. In short, given the wide possibility of indoor as well as outdoor cultivation, tomatoes are currently one of the foods with the greatest potential for use as an edible vaccine.

### 5.4. Lettuce

Experiments focusing on lettuce (*Lactuca sativa*) plants expressing the B subunit of the thermolabile protein of *E. coli*, which is responsible for enteric diseases both in humans and animals, indicate that this vegetable may be a potential edible vaccine. In this experiment, approximately 2% of the total protein detected in the leaves corresponded to the antigen [119]. In 2005, lettuce expressing glycoprotein E2 of the classical swine fear hog pest virus was developed [72]. In Poland, transgenic lettuce plants that produce effects against hepatitis B virus are in the first phase of development [120]. Because this food is mainly consumed raw, it has the greatest potential to be used as an edible vaccine.

### 5.5. Rice

A study in 2007 showed that transgenic rice (*Oryza sativa*) plants expressing the B subunit of *E. coli* induce a considerable amount of antibodies against this subunit [121]. In the same year, transgenic rice expressing the VP2 antigenic protein from infectious bursitis was shown to induce an immune response in chickens [86]. In 2008, functional expression of HBsAg in rice seeds was confirmed by PCR and Southern blot analyses [122]. Furthermore, in 2008, transgenic rice was produced in parallel to express the B subunit of the *E. coli* thermolabile toxin using the bioballistic approach to transform the plant cells; the expression was verified by PCR [123]. World rice production for 2016/2017 is estimated to be 480 million metric tons, and China and India (the two countries with the largest populations in the world) will produce and consume almost half of that annual production [124]. Thus, any vaccine developed using this plant will have a huge impact on the public health systems not only of these two countries but also other nations where rice is an important part of the daily diet.

### 5.6. Carrots

Transgenic carrots (*Daucus carota*) expressing the B subunit from *E. coli* thermolabile toxin induced IgA and IgG production, and occurred at the intestinal and systemic levels in rats [125]. In 2010, the UreB subunit of *Helicobacter pylori* in transgenic carrots was reported to have potential use as a possible vaccine [126]. Carrots, along with *A. thaliana*, were also utilized in experimental edible vaccines for surface HIV antigen expression, and studies performed in rats showed more positive effects in treated animals compared to non-treated animals [81]. The utilization of carrots to treat HIV appears promising not only because carrots are healthy and delicious but also because the consumption of carrot-derived carotenoids increases lymphocytes, monocytes and other immune defenders in rats [127]. Thus, people with weakened immune systems might benefit from consuming this potentially edible anti-HIV vaccine. Studies in humans must be conducted to confirm the potential of these vaccines.

### 5.7. Soybeans

B subunit expression studies of *E. coli* thermolabile toxin were conducted in the soybean (*Glycine max*) endoplasmic reticulum, in which a total antigen level of up to 2.4% of the soy seeds’ total protein was obtained without producing any instability during seed drying for further processing treatment; moreover, oral consumption by rats led to increases in systemic IgA and IgG levels [82].

### 5.8. Alfalfa

In 1999, successful oral immunization was achieved against virulent foot-and-mouth disease (FMDV) in rats, providing the first evidence that long protein chains can be successfully produced using only raw extracts when sufficient plant quantities are utilized [128]. Transgenic alfalfa (*Medicago sativa*) expressing the antigen eBRV4 from VP4 of hog rotavirus (BVR) was subsequently used as an edible vaccine in a veterinary environment [129]. In 2005, transgenic alfalfa plants were developed to express hog pest virus glycoprotein E2 [72]. In 2009, transgenic alfalfa development was reported in which alfalfa expressed the σC protein from the capsid virus, which causes poultry infections. The same antigen was developed in other plants, for example, *A. thaliana* [86,130]. In another alfalfa study, Eeg95-EgA31 of *Echinococcus granulosus* was expressed. This protein was purified and was also delivered directly from the leaves to the target organism [131], confirming the huge potential of this plant for veterinary purposes. 

### 5.9. Corn

In 2012, transgenic corn (*Zea mays*) plants expressing rabies virus antigenic glycoproteins showed quite promising results as an edible vaccine for both humans and animals [13,85]. Promising results have been obtained in relation to the development of vaccines against transmissible gastroenteritis coronavirus (TGEV) in pigs [24,25,26]. Studies using transgenic corn as a vaccine showed that 50% of treated pigs developed diarrhea, in contrast to 75% of pigs not treated with the vaccine. The study concluded that the transgenic corn conferred partial protection to piglets against clinical disease and experimental challenge with the pathogen [25,26]. In other studies, oral feeding with transgenic corn expressing the fusion protein of the Newcastle disease virus (NDV) produced immunogenic effects and conferred protective immunity in poultry [26,132].

### 5.10. Papaya

A vaccine based on papaya (*Carica papaya*) fruit was produced in 2007 by expressing synthetic peptides in 19 transgenic papaya clones to combat cysticercosis caused by *Taenia solium*. This vaccine was tested in rats, and 90% of treated rats showed an immunogenic response [80]. These edible vaccines could provide sweet relief in both humans and pigs, the main two disease carriers, but have not been tested in these systems.

### 5.11. Quinoa

In 2012, an edible vaccine was developed by expressing the VP2 antigen from infectious bursitis virus in quinoa (*Chenopodium quinoa*). The vaccine was developed for poultry veterinary medicine [133].

### 5.12. Bananas

The expression of HBsAg has been reported in banana plants using four different expression cassettes (PHB, PHER, pEFEHBS, and pEFEHER). Expression was studied at various levels using PCR, Southern hybridization and reverse transcription PCR. The expression levels in the crop plants reached a peak of 19.92 ng/g, and the antigen was present in the leaves of the plant [134,135]. However, the use of this vaccine was rejected due to the long periods of time that it takes for the tree to develop.

### 5.13. Peas

This transgenic plant was developed based on the expression of a capsid protein of Norwalk virus. Protein accumulation of up to 8% of the soluble protein was observed in the unripened fruit, with lower accumulation in red ripened fruits [98,115]. Expression in plant seeds allowed storage of the antigenic peptide and thus generated a plant with a high yield of protein expression; the protein content was estimated at 20% to 40% [98,115], and thus extraction of the pharmaceuticals would not require extensive purification procedures [98].

Pea plants have also been used for expression of the hemagglutinin protein (H), a PA against rinderpest virus. The level of expression was determined by Western Blot and was observed to be between 0.12% to 49% of the total soluble protein in leaves [98,136]. Thus, further studies to optimize protein expression in transgenic peas are needed.

### 5.14. Apples

The gene encoding the F protein of human respiratory syncytial virus (RSV)-F was constitutively expressed in apple leaves using the CaMV35S promoter. Protein expression was considered stable and corresponded to 20 mg/g of plant tissue [137].

### 5.15. Cherry Tomatillos

Lines of transgenic cherry tomatillos were developed for the HBsAg gene of hepatitis B. Gene expression was observed throughout the plant but was highest in the leaves, reaching 300 ng/g fresh weight, with 10 ng/g fresh weight in fresh fruit. Significant immune system activation was observed in rodents [138].

### 5.16. Algae

The green alga *Chlamydomonas reinhardtii* has been used as a model to produce large amounts of proteins related to therapeutic processes in both humans and animals [139]. The use of algae for vaccine production seems promising because algae have a very fast growth rate, their entire structures can be used as a raw material to produce edible vaccines, and there are no limitations in terms of habitat (sea farms) or aspects related to fertility [140]. Moreover, there are no negative concerns about cross-contamination with other field crops [102]. Additionally, algae can be cultivated in bioreactors [141] to further accelerate their already fast growth. Importantly, algal vaccine effectiveness is unaltered after lyophilization, which may facilitate the global distribution of edible vaccines made from algae [142,143]. In particular, the model alga *C. reinhardtii* contains only one chloroplast, increasing the stability of algal lines expressing the desired antigens [144].

The first report of recombinant proteins produced in algae described the expression of both the viral structural protein VP1 from foot-and-mouth disease virus and the β-subunit of cholera toxin (CTB) [29]. Superior results were obtained compared with previous expression in plants and testing in mice by Wigdorovitz et al. [128]. A second report established the in vivo efficacy of algal immunity for the first time. Specifically, the surface protein E2 of swine fever (CSFV) disease was expressed in the *C. reinhardtii* chloroplast genome, and the isolated proteins provoked an immune response after injection into swine. Unfortunately, no results of this assay were shown [102,145]. Other antigens, including glutamic acid decarboxylase (a known autoimmune agent of diabetes), the E7 protein of HPV, different fragments of proteins associated with *Plasmodium* (the agent that causes malaria), a surface antigen of hepatitis B and a protein from the virus that causes white spot syndrome, were also subsequently expressed in algae [102].

## 6. Current and Future Challenges

### 6.1. Current and Future Regulation of Edible Vaccine Production, Commercialization, and Copyright

As the primary research and reporting entity, the World Health Organization (WHO) assembled an Expert Board to discuss the scientific basis for the regulation of human candidates for vaccines derived from plants [146]. They concluded that the development of current vaccine lines and the evaluation and use of vaccines obtained in the traditional fashion could also be applied to vaccines derived from plants, although some specific topics related to production and waste have yet to be addressed [147]. In 2005, the World Health Organization (WHO) delivered a report on the implementation of good agricultural practices for the development of biopharmaceuticals. This report includes detailed information about methods of quality control for medicinal plants, testing to assess identity and purity, and recommended materials for plants in biopharmaceuticals [73,146]. It is important to highlight that vaccines that are derived from current plants are being produced and clinically tested according to the United States Investigational New Drug Research Application standards and good agrarian practices. This study has been sponsored by the United States Agriculture Agency (USDA), the European Medicine Agency (EMA), and the Cuba Regulatory Authority and has been announced at plant production conferences on antibodies and vaccines in France in 2004 and in the Czech Republic in 2005 [55]. The USDA has approved vaccines used in the veterinary field after reviewing and identifying the nature of the plant, the likelihood of cross-contamination, and the genetic background of the plants used as a vaccine. The USDA is also responsible for considering risks, taking into account physical and geographical aspects and plant reproduction [73]. The EMA published a report in 2008 on the importance of the quality of biologically active substances used for stable transgene expression in plants; this report mainly refers to plants that stably express the transgene and does not include transient expression in plants. Nonetheless, this report provides interesting ideas on the regulation of plant vaccines [73].

Because edible vaccines may be considered both food and medicine for some countries, regulatory and research institutions such as the United States Food and Drug Administration (FDA) have been seeking to rectify this duality to evaluate and regulate edible vaccines as a combined product. Furthermore, as a result of their physical features, edible vaccines are not suitable for regulation by other means, as in the case of additives and pharmaceutical products. If a genetically modified product is considered substantially equivalent to its natural counterpart, both are treated according to good harvest and safety practice regulations. The antigen that is present in edible vaccines is considered a chemical that does not comply with FDA rules concerning nutritional additives but is recognized as non-GRAS (Generally Recognized As Safe). Nevertheless, these vaccines, under the category of food, would be included as a genetically modified food and thus are not considered a high health risk [148].

Due to this ambiguity, a legal void currently exists with respect to regulations for standardizing edible vaccine commercialization. It is not yet clear what part of the vaccine discharges the antigen itself: the transgenic modified fruits or the transgenic seeds [149]. In the presence of this legal uncertainty, every country is expected to evaluate whether the entrance of edible vaccines (or the plants producing them) is permitted. 

Another remaining challenge to be overcome is identifying the most efficient method of vaccine production, i.e., agroinfection, viral vectors, or another method that generates a constant antigen concentration in the plant tissue, thus allowing large-scale production of the vaccine in question. The optimal method will depend on the containment and culture methods required to generate large quantities of a vaccine of this type. Among the methods mentioned above, the use of viral vectors is seen as the method that has the greatest potential. However, the use of viral DNA, even deconstructed (second generation of viral vectors) does not rule out the possibility that some mutation events occur that totally or partially reestablish the infectious capacity of the virus. However, this possibility seems remote since the only study that reported an apparent stimulation of the immune system by the consumption of a plant virus (Pepper mild mottle virus), lacked a clinical demonstration confirming that the detected symptoms corresponded to a pathogenic trait induced by this virus in the evaluated patients [150].

On the other hand, the development of edible vaccines will inevitably lead to the development of transgenic plants. As long as the studies are carried out on a laboratory scale, containment measures to avoid the risk of transgene escape into the environment would be relatively well covered by current biosafety standards. However, the development of large-scale “edible vaccines” would involve containment measures that would address not only the potential transfer of genes by hybridization to other wild plants, or the dispersal of pollen, but also the action of anti-transgenic and/or anti-vaccination activists. Furthermore, consideration should also be given to avoid theft of such foods, which could generate a problem of clandestine consumption where non-regulated ingestion dosage could lead to possible intoxication in the population.

Another important issue mentioned above is related to the cost of vaccine production. In this scenario, if costs are not reduced, the use of edible vaccines will be restricted. Therefore, to effectively produce an antigen in a plant or plant tissue, to determine the effective formulation of the vaccine including the correct adjuvants, and to establish an immunization regimen are three major challenges that need to be solved prior to the application of edible vaccines in the clinical field.

Despite these restrictions, the current view regarding edible vaccines remains positive in that they represent an important scientific development opportunity in the search for alternatives to immunize the population [151]. The potential of longer shelf-life compared to traditional vaccines and the vegetable origin of their coating matrix will facilitate mobilization of antigens from the mouth to the intestines triggering the immune response there (Section 3, Figure 1). These are two important factors that highlight the interest continuing to develop this type of non-traditional immunization system.

### 6.2. Ethical Aspects

The use of edible vaccines certainly raises ethical concerns, and one important concern is whether the edible vaccines are consumed as foodstuff or medicines. Such vaccines will continue to be regarded as genetically modified organisms, a term that alarms the majority of the population. For example, a study performed in Malaysia showed that, although such vaccination might be a less expensive way to vaccinate and prevent diseases, it was rejected outright due to the lack of familiarity with the topic. Another reason for this rejection is religious beliefs, which encourage the perception that transgenic organisms are a risk to society. In the realm of religion, a transgenic combination of species is a complete deviation [152]. Moreover, one of the main problems related to the generation of biotechnological tools is their perception as destructive and non-productive, as in the case of bioterrorism, which is defined as the threat of use or use of a biological agent by individuals or groups based on political, religious, ecological, or ideological goals [153]. Although genetically modified organism development has been under stringent control with regard to the acceptance of production and distribution, edible vaccines still require more regulation. Moreover, because edible vaccines represent a very powerful technological tool, their possible use by terrorists has not been discounted, and it remains difficult to eliminate such ill-intentioned use. However, edible vaccines could be used not only in an undesirable way but also as a solution to counter bioterrorism. An example is the edible vaccine developed in 2014 to combat anthrax (carbuncle), based on a tobacco plant expressing the PA; the antigen was tested using murine models that exhibited a high serum content of IgA and IgG [114]. Correspondingly, the possible beneficial or harmful use of current biological tools will depend on the people who develop them and the manner in which their use is regulated. Moreover, despite the future promise of edible vaccines, both veterinary and human medicine studies are lacking to promote their use within these areas and to restrict their ill-intentioned use.

### 6.3. Bioterrorism

The inappropriate management of a modified plant or fruit that contains a vaccine, whether due to negligence or ill-intentioned purposes (bioterrorism), would present serious challenges to public health and global safety. Consequently, countries must promptly discuss the advantages and disadvantages of developing new vaccination methods, their risks and benefits, and the regulation of edible vaccine cultivation, commerce, and distribution. The development of vaccines from genetically modified plants may be a key step in the development of strategies to address bioterrorism, as has been shown with Ebola virus surface glycoproteins, which can be transiently expressed in plants. This technology has been developed in conjunction with the U.S. Army to confront bioterrorism situations [154].

## 7. Conclusions

Edible vaccines represent a valuable solution to treating certain diseases whose control and prevention is restricted by the inherent limitations of traditional vaccines, such as their production costs, storage requirements, and expensive logistics. Sixteen foods are already producing antigens to counter human and animal diseases. However, some challenges remain, such as the development of edible vaccines using plants whose genetic transformation is difficult to attain or is unexplored, whose cultivars can be developed on all continents with low water and nutritional requirements, and whose consumption may be accomplished in a raw form or with minimal boiling. Because vaccine legal regulations are devoid of bylaws, many uncertainties would arise if such distribution were to gain acceptance. For example, who will be in charge of assigning the correct dose? As a drug that is contained in a plant or its fruit, should it be evaluated, authorized, and supervised by Public Health Institutes or a similar Human Health Organization in each country? These vaccines have undeniable potential to counter hundreds of diseases, particularly in countries where traditional vaccines are difficult to obtain or where the frequency of outbreaks of certain diseases makes their control and prevention more difficult. Beyond the pros and cons of edible vaccines, one of the most complex problems to address is the establishment of collaborations for the development of a stable vaccine that can actually be used in human medicine. Advances in the development of transgenic plants and antigen expression for stimulation of the immune system associated with the mucosa have been in the botanical field and not in immunology. As explained in the previous sections, it is very difficult to establish a stable antigenic protein concentration in plant tissues, and there is no certainty that the expressed antigen will produce an immune response. Efforts by immunologists and conventional vaccine developers could be of great value to advance this alternative to current vaccines. In addition to their possible benefits, edible vaccines will decrease the costs of vaccination and allow minimally invasive vaccine administration. Furthermore, reiterating the need to increase vaccine performance and stability, developments in the generation of transient vaccines using viruses do not obviate the development of transgenic plants as a long-term and longer-lasting measure. The potential opposing role of oral tolerance might be beneficial for the treatment of autoimmune diseases in which dendritic cells play a fundamental role in regulating and maintaining the balance between immunity and tolerance [155,156]. However, it is also necessary to discuss the potential global consequences of the inappropriate use of edible vaccines, particularly with respect to ecosystem imbalance (pollen and seed flight) and disturbances of worldwide peace and safety.

In summary, to reduce outbreaks of infectious diseases worldwide, the implementation of control and prevention measures on a massive scale is required. In this scenario, edible vaccines represent a valuable alternative to mitigate and prevent infectious outbreaks in countries where the conventional vaccination is difficult. In addition, in countries where the prevalence of infectious diseases is controlled, edible vaccines may support public health programs to reduce the risk of disease outbreaks, analogous to the use of prebiotics and probiotics as a complement to food. As shown in this work, the current production of edible vaccines is focused on a small group of plants, some of which are consumed globally. However, promoting the genetic transformation of plants with higher impact on the consumption chain in specific countries remains challenging. In addition, increasing the agricultural products of each country must be based on a consideration of country-specific policies with respect to the production or commercialization of genetically modified plants as well as ecological and cultural regulations, especially in those countries considered centers of origin of some important crops.

## Figures and Tables

**Table 1:** Onset of outbreak of infectious diseases around the world over the last six years (until September 2016) according the World Health Organization (WHO) [15,16,17,18]. Data are presented by continent, country, disease, and year of outbreak.

Infectious Diseases	Number of Countries Affected	Year(s) of Outbreak Occurrence (Since 2010)	Edible Vaccines Already Tested in Animals (Not Humans)
Zika	29	2015, 2016	
Poliomyelitis	19	2010, 2011, 2013 to 2016	
Measles	17	2010, 2011, 2013 to 2015	
Coronavirus (MERS-CoV)	15	2012 to 2016	Tomato [23], Corn [24,25,26]
Ebola	12	2011, 2012, 2014, 2015	
Yellow fever	12	2010 to 2013, 2016	
Cholera	8	2010 to 2013, 2015	Potato [27], Tomato [28], Algae [29]
Lassa fever	7	2012, 2015, 2016	
Chikungunya	6	2014, 2015, 2016	
Dengue	5	2010, 2012, 2015, 2016	
Avian influenza, H5N1 virus	5	2010 to 2014	
Rift Valley fever	4	2010, 2012, 2016	
West Nile virus	3	2011, 2014, 2015	
Microcephaly	3	2015, 2016	
Meningococcal disease	2	2010, 2015	
Plagues (bubonic, pneumonic)	2	2010, 2015	
Rubella	2	2014, 2015	Tomato [23]
Monkeypox	1	2016	
Marburg hemorrhagic fever	1	2012	
Typhoid fever	1	2015	
Hantavirus	1	2012	
Enterovirus D68	1	2014	
*Elizabethkingia*	1	2016	
Oropouche virus	1	2016	
Avian influenza, H7N9 virus	1	2013 to 2016	
Avian influenza, H5N6 virus	1	2014 to 2016	
Crimean-Congo hemorrhagic fever	1	2010	
Hemolytic uremic syndrome	1	2011	
Diphtheria	1	2015	
Enterohemorrhagic *Escherichia coli*	1	2016	

**Table 2:** List of plants studied as edible vaccines. The checklist is organized by year, since 1998 until today.

Year	Plant	Disease or Infectious Agent	Antigen	References
1998	Potato	Enteritis produced by *Escherichia col*	-	[103]
1998	Potato	Norwalk virus capsid	-	[104]
1998	Potato	Non-toxic subunit (CT-B) of *Vibrio cholerae* enterotoxin	-	[27]
1998	Potato	Rabbit hemorrhagic	Protein VP60	[106]
2003	Algae	Foot-and-mouth disease virus	Viral structural protein VP1	[29]
2003	Cherry tomatillo	Hepatitis B	HBsAg (surface protein of Hepatitis B)	[138]
2003	Pea	Rinderpest virus	Hemagglutinin protein (H)	[98,136]
2004	Alfalfa	Hog rotavirus (BVR)	Antigen eBRV4	[129]
2005	Banana	Hepatitis B	HBsAg (surface protein of Hepatitis B)	[134,135]
2005	Lettuce	Hog pest virus	Glycoprotein E2	[72]
2005	Potato	Hepatitis B	-	[72]
2005	Tomato	Coronavirus	-	[23]
2006	Tomato	Norwalk virus	Surface protein	[50,115]
2007	Algae	Swine fever (CSFV) disease	Surface protein E2	[102,145]
2007	Papaya	Cysticercosis caused by *Taenia solium*	Synthetic peptides	[80]
2007	Rice	Infectious bursitis	VP2 protein	[86]
2007	Tomato	*Vibrio cholerae* B toxin	CT-B protein	[28]
2007	Tomato	Hepatitis B	HBsAg (surface protein of Hepatitis B)	[50,116,117]
2007	Tobacco *	Chicken infectious anemia	Virus VP1 protein	[109]
2008	Rice	Hepatitis B	HBsAg (surface protein of Hepatitis B)	[122,123]
2010	Carrot	*Helicobacter pylori*	Subunidad UreB	[126]
2010	Corn	Rabies virus	Antigen glycoproteins	[13,85]
2012	Tobacco *	Avian flu virus	HPAIV H5N1	[112,113]
2012	Quinoa	Infectious bursitis virus	VP2 protein	[133]
2014	Algae	Diabetes	Glutamic acid decarboxylase	[102]
2014	Algae	Human Papilloma Virus	E7 protein	[102]
2014	Algae	Hepatitis B	HBsAg (surface protein of Hepatitis B)	[102]

* Although the tobacco plant is not a food, we have included it because it has been demonstrated that it can serve as a pharma plant.

## Supplementary Materials

The following is available at www.mdpi.com/2076-393X/5/2/14/s1, Table S1: Onset of outbreaks of infectious diseases around the world over the last six years (until September 2016), according to the World Health Organization (WHO) [15,16,17,18]. Data are presented by continent, country, disease, and year of outbreak.
