# Supplementary material for: Disease Prevention: An Opportunity to Expand Edible Plant-Based Vaccines?
Source: Vaccines (Basel). 2017 May 30;5(2):14. doi: 10.3390/vaccines5020014 (PMC5492011; doi:10.3390/vaccines5020014)
Supplement: Supplementary file 1 [file vaccines-05-00014-s001.pdf]

Table S1. Onset of outbreak of infectious diseases around the world over the last six years (until September 2016), according to the World Health Organization (WHO) [14–17]. Data are presented by continent, country, disease, and year of the outbreak.

Continent	Country (or Geographical Region)	Disease and/or Epidemiological AGENT	Month and Year that the Outbreak Occurs
Africa	Central Africa	Cholera	October 2010
		Poliomielitis	June 2014
		Monkeypox	October 2016
	West Africa	Ebola virus	April, May, June, July, August, September 2014
	Angola	Poliomielitis	July, September, 2010
		Yellow fever	February, March, April, June 2016
	Benin	Lassa fever	February, June 2016
	Burkina Faso	Dengue Fever	November 2016
	Cabo Verde	Zika virus	December 2015
	Cameroon	Yellow fever	February and May 2010
			February 2012
			October 2013
		Wild Poliovirus	November 2013
		Poliomielitis	March 2014
	Ivory Coast	Yellow fever	January 2010.
			January 2011.
	Horn of Africa	Wild Poliovirus	May, October, 2013
	Chad	Meningococcal disease, *Neisseria meningitidis* infectious agent	April 2010
			March 2011
		Poliomielitis	June 2011
		Yellow fever	February 2013
	Egypt	Dengue	November 2015
	Guinea	Yellow fever	January 2010
		Ebola hemorrhagic fever	March 2014
	Ghana	Yellow fever	February 2012
	Kenya	Chikungunya	August 2016
	Liberia	Ebola hemorrhagic fever	March 2014
		Lassa fever	May 2016
	Madagascar	Poliovirus	July 2015
		Plague (bubonic, pneumonic and septicemic)	September 2015
	Mali	Ebola virus	October 2014
	Mauritania	Rift Valley fever	November 2012

	Nigeria	Lassa fever	April 2012
			January, May 2016
		Meningococcal disease	March 2015
		Rift Valley fever	September, November 2016
		Poliovirus	October 2016
	Democratic Republic of Congo	Yellow fever	July 2010
			December 2012
			April, June 2016
		Poliomielitis	September, November 2010
		Cholera	July 2011
			July 2012
			December 2015
		Ebola hemorrhagic fever	August, September, October 2012
	Senegal	Yellow fever	October 2010
			December 2011
		Ebola virus	August 2014
		Chikungunya	September 2015
	Sierra Leone	Yellow fever	March 2011
		Cholera	August, September, October 2012
		Ebola virus	June 2015
	South Africa	Rift Valley fever	May 2010
	Sudan	Yellow fever	November, December 2012
			December 2013
	South Sudan	Hemorrhagic fever syndrome	May 2016
	United Republic of Tanzania	Cholera	September, October, November 2015
	Togo	Lassa fever	March 2016
	Uganda	Yellow fever	January 2011
			April 2016
		Ebola hemorrhagic fever	May 2011
			July, August, November 2012
		Marburg haemorrhagic fever	October, November 2012
			October 2014
		Typhoid fever	March 2015
America	Argentina	Zika virus	March 2016
		Chikungunya	March 2016
	Barbados	Zika virus	January 2016
	Bolivia	Zika virus	January 2016
	Brazil	Poliovirus in sewage	June 2014

		West Nile Virus	November 2014
		Zika virus	October 2015
		Microcephaly	November, December 2015
			January 2016
		Guillain-Barré syndrome	February 2016
	Colombia	Zika virus	October 2015
		Guillain-Barré syndrome	February 2016
	Cuba	Zika virus	March 2016
	Chile	Measles	March, April, June, 2011
			May, June 2015
		Zika virus	April 2016
	Ecuador	Zika virus	January 2016
	El Salvador	Zika virus	November 2015
		Guillain–Barré syndrome	January 2016
	United States of America	Hantavirus pulmonary syndrome	September 2012
		Enterovirus D68	September 2014
		Ebola virus	October 2014
		Measles	January, August 2013
			December 2014
			January, most wanted, March, April, May 2015
		Lassa fever	May 2015
		Microcephaly	February 2016
		Zika virus	February 2016
		Guillain–Barré syndrome	March 2016
		Elizabethkingia	April 2016
		Chikungunya	June 2016
	Guatemala	Zika virus	November 2015
	French Guiana	Zika virus	January 2016
	Haiti	Cholera	October, November 2010
		Zika virus	January 2016
	Honduras	Zika virus	December 2015
	Mexico	Cholera	October, November 2013
		Zika virus	December 2015
	Panama	Zika virus	December 2015
		Microcephaly	March 2016
		Guillain-–arré syndrome	March 2016
	Paraguay	Zika virus	December 2015
	Peru	Plague (bubonic, pneumonic and septicemic)	August 2010

		Zika virus	April 2016
		Oropouche virus disease	June 2016
	Puerto Rico	Zika virus	January 2016
	Dominican Republic	Zika virus	January 2016
	Saint Lucia	Zika virus	April 2016
	Surinam	Zika virus	November 2015
	Trinidad and Tobago	Zika virus	February 2016
	Uruguay	Dengue	March 2016
	Venezuela	Zika virus	December 2015
		Guillain–Barré syndrome	February 2016
Asia	Saudi Arabia	Middle East respiratory syndrome coronavirus (MERS-CoV)	May, June, July, August, September, October, November, December 2013
			January, February, March, April, May, June, July, October, November, December 2014
			May, June, July, August, September, October, November, December 2015
			January, February, March, May, June, July, September, October, November 2016
	Central Asia	Poliomyelitis	November 2010
	Bangladesh	Avian influenza, H5N1 virus infectious agent	March 2011
			March 2012
	Cambodia	Avian influenza, H5N1 virus infectious agent	February, April, August 2011
			January, April, May 2012
			February 2013
	China	Avian influenza, H5N1 virus infectious agent	May, November 2010
			January, June 2012
			January 2014
		Wild poliovirus	September 2011
		Avian influenza, H7N9 virus infectious agent	April, May, July, August, October, November, December 2013
			January, February, March, April, May, June, September, October, December 2014
			March, June, July, October, November, December 2015
			January, February, March, May, July , August, November 2016
		Avian influenza, H5N6 virus infectious agent	December 2014
			July 2015
			January, March, May, June 2016
		Measles	Throughout 2014

			January, February, March, April 2015
		Rubella	May, June, July, August, October, November 2014
			January, March 2015
		Middle East respiratory syndrome coronavirus (MERS-CoV)	May 2015
		Yellow fever	April 2016
		Rift Valley fever	August 2016
	United Arab Emirates	Middle East respiratory syndrome coronavirus (MERS-CoV)	May, June 2015
			January 2016
	The Philippines	Measles	Throughout 2014
			January, February, March 2015
		Middle East respiratory syndrome coronavirus (MERS-CoV)	July 2015
	Indonesia	Avian influenza, H5N1 virus infectious agent	February, July, August, October, December, 2010
			March, April, May, June, October, November 2011
			January, February, March, April, July, 2012
	Iraq	Cholera	September, October, November 2015
	Iran	Middle East respiratory syndrome coronavirus (MERS-CoV)	May 2015
	Israel	Poliovirus	August, September 2013
	Japan	Measles	Throughout 2014
			January, February, March, April 2015
	Jordan	Middle East respiratory syndrome coronavirus (MERS-CoV)	September, October 2015
	Kuwait	Middle East respiratory syndrome coronavirus (MERS-CoV)	September 2015
	Kyrgyzstan	Measles	January 2015
	Malaysia	Measles	Throughout 2014
			January, February, March, April 2015
	Maldives	Zika virus	January 2016
	Mongolia	Measles	March, April 2015
	Oman	Middle East respiratory syndrome coronavirus (MERS-CoV)	June 2015
			January 2016
	Pakistan	Crimean–Congo hemorrhagic fever	October 2010
		Dengue	October 2010
		Cholera	October 2010
		The international spread of wild poliovirus from Pakistan is confirmed	September 2011
	Qatar	Middle East respiratory syndrome coronavirus (MERS-CoV)	October 2014
			May 2015

			March, May, June 2016
	Republic of Korea	Middle East respiratory syndrome coronavirus (MERS-CoV)	May, June, July, October, November 2015
		Measles	April, May, June, July, August, September, October 2014
			March 2015
	Myanmar	Poliovirus	December 2015
	Lao People's Democratic Republic	Poliovirus	November, December 2015
			January 2016
	Russia	Poliomyelitis	November 2010
	Singapore	Measles	April, May, June, July, August, October, December 2014
			January, February 2015
	Syria	Poliomyelitis	October, November 2013
			March 2014 (worldwide spread)
	Thailand	Middle East respiratory syndrome coronavirus (MERS-CoV)	June, July 2015
			January, August 2016
	Tajikistan	Poliomyelitis	April 2010
	Vietnam	Avian influenza, H5N1 virus infectious agent	March, April 2010
			January, February, March 2012
		Measles	Throughout 2014
			January, February, March 2015
		Zika virus	April 2016
Europe	Austria	Middle East respiratory syndrome coronavirus (MERS-CoV)	September 2016
	Germany	Hemolytic uremic syndrome outbreak	May, June 2011
		Middle East respiratory syndrome coronavirus (MERS-CoV)	March 2015
		Lassa fever	March 2016
	Europe	West Nile virus (WNV)	August 2011
		Measles	March 2015
	Spain	Ebola virus	October 2014
		Measles	November 2010
			January, August 2011
			June, July 2013
			January, February, March, July 2014
		Diphtheria, infectious agent *Corynebacterium difteriae*	May, June 2015
		Chikungunya	August, September 2015
	France	Measles	January, February, March, April 2011
		Chikungunya	October 2014
		Zika virus	March 2016

	Portugal	Dengue	October 2012
		West Nile Virus	September 2015
	United Kingdom	A new coronavirus infection	September, October, November 2012
			February, March, May, 2013
		Ebola virus disease	December 2014
		Enterohaemorrhagic *Escherischia coli*	July 2016
	Sweden	Lassa fever	April 2016
	Turkey	Middle East respiratory syndrome coronavirus (MERS-CoV)	October 2014
Oceania	Australia	Measles	Throughout 2014
			January, February, March, April 2015
	New Zealand	Measles	April, May, June, July, August, October, November 2014
			February 2015
		Rubella	June 2014
	Papua New Guinea	Measles	Throughout 2014
			January, February, March 2015
		Zika virus	April 2016
